# Lp-PLA2 as a biomarker and its possible associations with SARS-CoV-2 infection

**DOI:** 10.2217/bmm-2021-1129

**Published:** 2022-06-13

**Authors:** Pamila Dua, Archana Mishra, KH Reeta

**Affiliations:** ^1^Department of Pharmacology, All India Institute of Medical Sciences, Ansari Nagar, New Delhi, 110029, India

**Keywords:** biomarker, COVID-19, Lp-PLA2

## Abstract

Lp-PLA2 is an enzyme encoded by the *PLA2G7* gene located at chromosome 6p12-21, which is included in different signal transduction pathways. The potential of serum levels of Lp-PLA2 as a marker of inflammation quantifying cardio-metabolic risk, renal impairment and oxidative stress has been explored in earlier studies. It has also been used in chronic obstructive pulmonary disease, hepatic disease, metabolic conditions and exercise tolerance. Additionally, it shows promising evidence for the assessment of risk for certain cardiovascular conditions in otherwise seemingly healthy individuals. COVID-19 has affected life and the economy globally. The identification of biomarkers to assess the sickness and treatment plan is the need of the hour. This review summarizes the pathophysiological inter-relationship between serum levels of Lp-PLA2 and COVID-19. The authors hypothesize that the estimation of Lp-PLA2 levels may help in the early identification of risk and thus may play a beneficial role in the proactive management of COVID-19.

## Graphical abstract

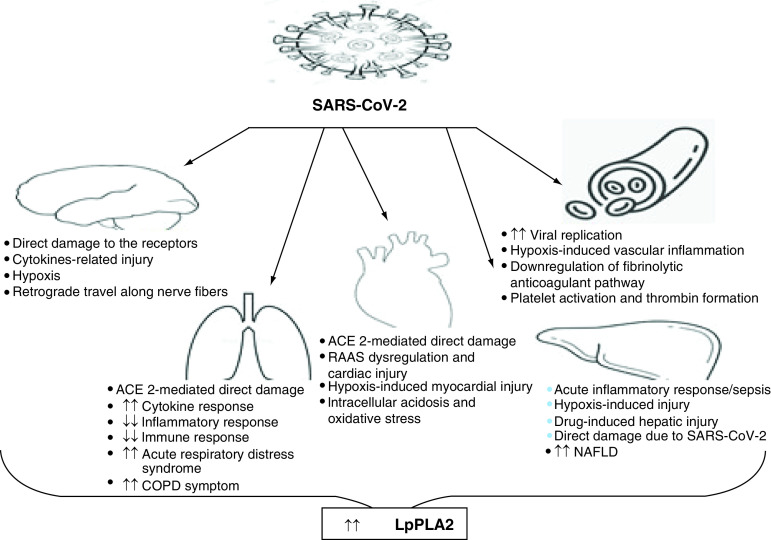

COVID-19 caused by SARS-CoV-2 has affected millions of people in more than 200 countries. It was declared a global public health emergency by the WHO in early 2020. A mortality rate of 1–10% for mild to severe disease has made a grim picture. As of 23 December 2021, the total number of confirmed cases was reported to be more than 275 million, with the number of deaths around 5 million [1]. Although the number of cases shows a trend of waxing and waning, severe disease and mortality still exist due to the emergence of mutant variants of the virus. Needless to say, SARS-CoV-2 poses a great threat to public health globally, and fatalities are more prevalent in patients with comorbidities.

Previous studies have shown that the most common comorbidities in patients with COVID-19 are cardiovascular diseases. Most of the hospitalized patients with COVID-19 already had diabetes, hypertension, other cardiac diseases, renal disease, chronic obstructive pulmonary disease or previous cerebrovascular accidents; a few had cancer, and some had been infected with HIV. A significant number of patients suffered from multiple conditions [2]. Despite the disease persisting for 2 years at the time of writing, its pathophysiological picture is still not very clear. Clinicians and researchers have been diligently trying to establish a laboratory test panel to capture the disease and assess the severity and prognosis. This includes routine hematological parameters (e.g., total white blood cells, natural killer cells, lymphocytes, neutrophils, platelets, eosinophils, T cells and B cells), biochemical markers of liver function (e.g., total bilirubin, alanine aminotransferase and aspartate aminotransferase) and kidney function tests (e.g., blood urea, creatinine and lactic acid dehydrogenase [LDH]), as well as inflammatory markers (e.g., erythrocyte sedimentation rate, C-reactive protein, interleukins such as IL-6, IL-2R, IL-8 and IL-10 and TNF-α) and coagulation function indices (e.g., prothrombin time, fibrinogen, D-dimer, high-sensitivity troponin and brain natriuretic peptide). Some studies have used biomarkers such as homocysteine, angiotensin II, neutrophil–lymphocyte ratio and monocyte–lymphocyte ratio, to name a few [3–6]. The oxygen saturation index has been used as a good indicator for stratifying patients with regards to the severity of COVID-19. The current target oxygen saturation range to avoid hospitalization for patients with COVID-19 is 92–96%, as recommended by the NIH [7]. However, none of these tests are definitive; hence a constant search for new biomarkers for the diagnosis and prognosis of the disease is ongoing. Moreover, monitoring an extra-large panel of laboratory tests imposes a great economic burden and would be a waste of time. The availability of reliable and validated markers to distinguish severe from non-severe conditions will indeed be helpful in triaging and treating patients, especially in developing countries with limited healthcare resources.

Lp-PLA2, originally named plasma platelet activating factor acetyl-hydrolase, is an enzyme encoded by the *PLA2G7* gene located at chromosome 6p12-21, and is included in different signal transduction pathways. Lp-PLA2 is one of the members of the super family of PLA2 enzymes. The molecular weight of Lp-PLA2 is 468.4 g/mole and it is composed of 441 amino acids. The morphology of Lp-PLA2 in atherosclerotic plaque is somewhat different from that of normal circulation. The normal range for Lp-PLA2 is less than 200 ng/ml in healthy individuals [8]. It is secreted by the macrophages and circulates in the blood in the form of a complex with lipoproteins ~70% with low-density lipoprotein (LDL) and ~30% with high-density lipoprotein (HDL) [8]. Additionally, it can switch between these two lipoprotein subtypes. Lp-PLA2 hydrolyzes oxidized phospholipids to pro-inflammatory products, which are the key factors in endothelial dysfunction, plaque inflammation and the development of necrotic core in the plaque [9]. It has been found to be associated with the oxidative modification of LDL, as well as the development of an inflammatory cascade in the arterial intima. The potential of serum levels of Lp-PLA2 to be a marker of inflammation quantifying cardio-metabolic risk [10], renal impairment [11] and oxidative stress has been studied [12]. A meta-analysis of 32 prospective studies concluded that Lp-PLA2 levels are positively correlated with the risk of coronary heart disease and stroke [13]. Increased oxidative stress leads to increased production of pro-inflammatory cytokines and *vice versa*, and thus a vicious cycle ensues, which increases the severity of the cardio-metabolic environment [14]. The European Society of Cardiology has recommended the estimation of Lp-PLA2 levels as one of the predictors for the recurrence of cardiac events [15]. The American Heart Association/American Stroke Association has recommended the measurement of Lp-PLA2 levels in healthy individuals to identify the risk of stroke [16]. Lp-PLA2 has also been used to predict the incidence and progression of diabetic retinopathy [8]. A strong correlation has been reported between Lp-PLA2 and acute physiology and chronic health evaluation (APACHE II) scores, which are used to assess disease severity and mortality in critically ill sepsis patients [17]. Lp-PLA2 has also been found to be a promising biomarker for chronic obstructive pulmonary disease (COPD) and for the prediction of low exercise tolerance [18].

Based on genetic expression, several variations have been detected in terms of gain or loss of function at the level of the *PLA2G7* gene [19] that exhibit altered physiology and homeostasis, ultimately ending in different disease conditions. A large number of diseases that have been linked to the *PLA2G7* gene polymorphism are of cardio-cerebral origin, but asthma, schizophrenia, chronic kidney disease, multiple sclerosis, ulcerative colitis and Kawasaki illness have also been linked to *PLA2G7* polymorphisms [20].

COVID-19 has not been known to spare any organ and has an established notoriety for creating an exhaustive list of complications, such as venous thromboembolism, cardiovascular complications (e.g., myocarditis, heart failure, arrhythmias, acute coronary syndrome, rapid deterioration and sudden death) [21], acute kidney injury [22], liver injury [23], neurological complications [24] and respiratory failure [25]. COPD is also linked to an increase in COVID-19 severity and mortality [25].

Assessment of Lp-PLA2 levels in blood sample can be done with simple methods such as ELISA [26]. Thus, the measurement of Lp-PLA2 levels may serve as a simple yet valuable biomarker. In this article, the authors give a brief overview of the role of Lp-PLA2 in different disease conditions and attempt to connect the dots between COVID-19 and various organ system injuries.

## Lp-PLA2 in cardiovascular diseases

The pro-inflammatory role of Lp-PLA2 is due to lyso-phosphatidylcholine (lysoPC) and oxidized non-esterified fatty acids (oxNEFAs), which are hydrolysis products of oxidized LDL. These lysophospholipids induce inflammatory changes in multiple cells, including vascular endothelial cells, smooth muscle cells, monocytes/macrophages, T cells and neutrophils [27]. Lp-PLA2 also affects cell viability, induces oxidative stress and modulates immune responses [28]. LysoPC is responsible for processes such as apoptosis, the upregulation of adhesive molecules, the increased expression of interferon-γ, inflammatory mediators and growth factors. Furthermore, oxNEFAs cause increased cellular permeability and apoptosis, leading to increased oxidative stress and retention of LDL [29]. Lp-PLA2 circulates in the blood and its pro-inflammatory role has been suggested in many vascular diseases. Downregulation of the expression of the *PLA2G7* gene by RNA interference has also been shown to ameliorate inflammation and atherosclerosis in mice deficient in apo-lipoprotein E [30]. Lp-PLA2 is reported to play a crucial role in the development of atherosclerosis. Thus, Lp-PLA2 can serve as an important marker of plaque instability and inflammatory pathways leading to cardiovascular morbidities [31]. Studies assessing the diagnostic and prognostic functions of Lp-PLA2 in different cardiovascular diseases are listed in Table 1.

**Table 1.** Studies assessing the diagnostic and prognostic roles of Lp-PLA2 in cardiovascular disease conditions.

Study	Conclusion	Ref.
Younus *et al.*	Significant association of Lp-PLA2 with carotid intima-media thickness was observed.	[32]
Ahmed *et al.*	Clinical utility of Lp-PLA2 and its validity as an independent cardiovascular disease biomarker has been established.	[33]
Gerber *et al.*	Lp-PLA2 was independently related with mortality and risk prediction in cardiovascular disorders.	[34]
Möckel *et al.*	Lp-PLA2 was found to be a good marker for major adverse cardiac events, cardiac death, non-fatal myocardial infarction, unstable angina and congestive heart failure requiring admission for urgent coronary interventions.	[35]
Tsimikas *et al.*	Increased Lp-PLA2 was related with metabolic disorder and was affected by ferritin levels, low-density lipoprotein cholesterol and apolipoprotein B, suggesting its participation in lipid peroxidation.	[36]
Ikonomidis *et al.*	Elevated Lp-PLA2 concentration was associated with endothelial dysfunction, carotid atherosclerosis, impaired coronary flow reserve, elevated arterial stiffness and unfavorable consequence in coronary artery disease patients. These findings endorse the prognostic role of Lp-PLA2 in persistent coronary artery disease.	[37]
Li *et al.*	Lp-PLA2 was associated with major unfavorable cardiac events and risk score.	[38]
Koenig *et al.*	Increased concentrations of Lp-PLA2 anticipate future cardiovascular events in patients with cardiovascular disorders.	[39]
Möckel *et al.*	In patients with suspected acute coronary syndrome, N-terminal pro-brain natriuretic peptide, troponin I and Lp-PLA2 were predicted to be good markers for risk stratification.	[40]
Searle *et al.*	The diagnostic, prognostic and therapeutic value of Lp-PLA2 in the setting of acute coronary syndrome and percutaneous coronary intervention was established.	[41]
Krintus *et al.*	Lp-PLA2 may be considered to facilitate risk stratification in acute coronary syndrome patients and in healthy individuals with intermediate cardiovascular risk.	[42]

## Lp-PLA2 in cerebrovascular disorders

LysoPC has been found to be associated with loss of pericyte in the central nervous system, leading to a loss of integrity of the blood–brain barrier [43]. This leads to disruption of tight junctions and initiates inflammatory responses that enhance permeability and lead to the leakage of harmful substances from blood vessels into the brain. These events ultimately contribute to cerebral vascular disorders such as stroke, transient ischemic attack and Alzheimer's disease (AD) [44]. In neurovascular dysfunction caused by vascular inflammation, Lp-PLA2 has been shown to be related to disease progression and has been cited as an independent risk factor for dementia [45] and AD [46]. Additionally, Lp-PLA2 has been demonstrated as a reliable biomarker for Parkinson's disease [47]. Studies assessing the diagnostic and prognostic functions of Lp-PLA2 in different cerebrovascular diseases are listed in Table 2.

**Table 2.** Studies assessing the diagnostic and prognostic roles of Lp-PLA2 in cerebrovascular disease conditions.

Study	Conclusion	Ref.
Li *et al.*	A high serum Lp-PLA2 level was correlated with acute ischemic stroke incidence, disease severity and recurrence.	[48]
Delgado *et al.*	Significant changes in Lp-PLA2 concentrations occur early after stroke and may add relevant information in early arterial recanalization in intravenous tissue plasminogen activator-treated patients.	[49]
Han *et al.*	The elevated Lp-PLA2 mass was associated with all-cause death independently of other risk elements after acute ischemic stroke.	[50]
Bian *et al.*	Serum Lp-PLA2 was postulated as a potential prognostic biomarker of intra-cerebral hemorrhage.	[51]
Tsai *et al.*	Elevated Lp-PLA2 mRNA expression of peripheral blood mononuclear cells appeared to be a potential biomarker for predicting an unfavorable outcome in patients with acute ischemic stroke.	[52]

## Lp-PLA2 levels & oxygen saturation index

Oxygen saturation is calculated as the fraction of oxygen-saturated hemoglobin with respect to total hemoglobin (unsaturated + saturated) in the blood. The human body requires and regulates a very precise and specific balance of oxygen in the blood. Mean arterial blood oxygen saturation level in healthy human beings is maintained within 95–100%. Levels below 90% are considered harmful for proper ventilation of body tissues [7]. A few studies have shown that patients suffering from obstructive sleep apnea (OSA) have intermittent oxygen desaturation associated with periods of apnea or hypopnea. Therapy is targeted at the correction of apnea, to prevent hypoxemia. Oxygen desaturation is also associated with a high pro-inflammatory burden, and higher serum Lp-PLA2 levels have been observed in patients with oxygen desaturation in OSA syndrome. Studies in obese children with OSA showed increased plasma levels of Lp-PLA2 [53].

## Lp-PLA2 in COPD

Lp-PLA2 can regulate the immune pathway and inflammation through macrophages. In COPD, there is an accumulation of macrophages, leading to airway obstruction [53]. It has been reported that the increased expression of the *PLA2G7* gene in patients with COPD is associated with the severe airway obstruction measured through Global Initiative for Chronic Obstructive Lung Disease (GOLD) criteria. The 6-minute walk distance (6MWD) is a well-known clinical assessment parameter for patients with COPD [54]. However, a study by Deng *et al.* [55] found that Lp-PLA2 can be used as an easily assessed and accurate parameter for severity stratification when compared with 6MWD. Studies assessing the diagnostic and prognostic functions of Lp-PLA2 in COPD are listed in Table 3.

**Table 3.** Studies assessing the diagnostic and prognostic roles of Lp-PLA2 in chronic obstructive pulmonary disease.

Study	Conclusion	Ref.
Deng *et al.*	Lp-PLA2 was shown to be a promising biomarker for chronic obstructive pulmonary disease patients and suitable for assessing exercise tolerance in clinical practice.	[56]
Seri *et al.*	A correlation of superimposed thrombosis was shown in patients with respiratory infection and increased Lp-PLA2 levels as well as vascular endothelial growth factor.	[57]

## Lp-PLA2 in viral infections

Immunity is a natural, multifaceted process with the ability to identify and discriminate between self and foreign [58]. Macrophages and phospholipases are assumed to play a key role in immune activation and inflammation in viral infections such as HIV. Notably, patients with HIV exhibit increased arterial inflammation, which tends to persist even after effective antiretroviral therapy. Lp-PLA2 levels are observed to be unusually high in HIV-infected patients, and levels of the enzyme show direct correlation with risk factors associated with cardiovascular disease (CVD). Statin therapy in HIV-infected patients has been reported to reduce Lp-PLA2 levels, which further leads to a decrease in specific markers of immune activation and arterial inflammation, as well as non-calcified plaque volume. Therefore, Lp-PLA2 has been identified as a suitable predictor of subclinical atherosclerosis and can be a therapeutic target to prevent CVD in HIV-infected individuals [59]. Studies assessing the diagnostic and prognostic functions of Lp-PLA2 in viral infections are listed in Table 4.

**Table 4.** Studies assessing the diagnostic and prognostic roles of Lp-PLA2 in viral infections.

Study	Conclusion	Ref.
Srinivasa *et al.*	Comparative analyses among HIV-infected and non-infected individuals revealed significant relationships among subcutaneous adipose tissue, visceral adipose tissue and Lp-PLA2 levels.	[60]
Di Yacovo *et al.*	In naive HIV-infected patients, initiation of combination antiretroviral therapy resulted in an improvement in low-density lipoprotein particle phenotype and in the inflammatory/immune biomarkers, including Lp-PLA2.	[61]
Mabel *et al.*	Pitavastatin 4 mg daily vs pravastatin 40 mg when given for 52 weeks led to a greater reduction in markers of immune activation and arterial inflammation in terms of CD14 count, oxidized low-density lipoprotein and Lp-PLA2 level in patients with HIV.	[62]
Mayne *et al.*	HIV-associated inflammation was linked to increased Lp-PLA2, providing a mechanistic link between HIV and cardiovascular disease.	[63]
Ross Eckard *et al.*	Lp-PLA2 may represent a valuable early biomarker of cardiovascular disease risk in HIV infection before subclinical atherosclerosis can be detected.	[64]
Tsoupras *et al.*	HIV infection was associated with risk of AIDS, cardiovascular complications and a subsequent increase in Lp-PLA2.	[65]
Young *et al.*	Antiviral therapy in HIV-naive subjects resulted in decreased Lp-PLA2 levels along with IL-6 and high-sensitivity CRP.	[66]
Díaz-Pollán *et al.*	HIV-infected patients had higher Lp-PLA2 levels, and tobacco smoking was significantly associated with increased Lp-PLA2 levels.	[67]
Ross Eckard *et al.*	Statins may be helpful in attenuating cardiovascular disease risk in HIV-infected individuals by decreasing Lp-PLA2 levels.	[68]
Funderburg *et al.*	Tenofovir alafenamide and tenofovir disoproxil fumarate showed a good impact on immune activation and vascular inflammation in terms of Lp-PLA2 and other systemic markers, such as IL-6, high-sensitivity CRP, soluble tumor necrosis factor receptors and D-dimer.	[69]
Hileman *et al.*	Reduction in monocyte activation and vascular inflammation with integrase inhibitor-based initial antiretroviral therapy among HIV-infected individuals was associated with a decrease in Lp-PLA2 levels.	[70]
Huang *et al.*	Potential role of Lp-PLA2 as a prognostic biomarker in patients with sepsis during the early course of emergency intensive care unit treatment is established.	[71]

## Lp-PLA2 & coagulopathy

The trigger for inflammation following infection by a pathogen is release of various pathogen-associated molecular patterns and the consequent release of various cytokines, such as IL-6 and TNF-α. Eventually, it can cause disturbances in the integrity of the epithelial–endothelial barrier by recruiting circulating neutrophils and macrophages to the site of injury. Pro-inflammatory cytokines expose and upregulate the expression of tissue factors present in alveolar epithelial cells, macrophages, endothelial cells and fibroblasts present in blood vessel adventitia and platelets and cause the formation of clots by the activation of extrinsic pathway factors. Studies have shown that Lp-PLA2 activity affects LDL oxidation through lysoPC and indirectly acts as a marker in the early detection of the risk in terms of its associations with coagulation time [72].

## Lp-PLA2 in diabetes

Diabetes mellitus is one of the primary causes of illness and mortality worldwide, and its prevalence is expected to climb dramatically in the coming decades. Hyperglycemia has been well explained to cause inflammation that can lead to endothelial dysfunction and CVD [73]. Lp-PLA2 is related to inflammation; therefore, the possible inflammatory reaction mechanisms underlying diabetes can lead to increased levels of Lp-PLA2 [74]. In individuals with type 2 diabetes mellitus, higher serum levels of Lp-PLA2 were associated with a higher incidence of lower extremity arterial disease. It has been reported that inflammatory activities linked to the hydrolysis of oxidized phospholipids and the accumulation of platelet activating factor in adipose tissues are involved in the pathways, which could lead to increased insulin resistance [75]. Studies assessing the diagnostic and prognostic functions of Lp-PLA2 in metabolic syndrome are listed in Table 5.

**Table 5.** Studies assessing the diagnostic and prognostic roles of Lp-PLA2 in metabolic syndrome.

Study	Conclusion	Ref.
Persson *et al.*	Lp-PLA2 was related to the metabolic disorder. Higher plasma levels of Lp-PLA2 may increase the of cardiovascular disease in cases of metabolic syndrome.	[76]
Colak *et al.*	Serum Lp-PLA2 level was strongly related to histological steatosis scores in patients with non-alcoholic fatty liver disease.	[77]
Young *et al.*	Lp-PLA2 was correlated with metabolic pathways and fatty liver index.	[78]
Daskalopoulos *et al.*	Lp-PLA2 levels were significantly higher in women with polycystic ovary syndrome compared with lean controls.	[79]

## Lp-PLA2 in non-alcoholic fatty liver disease

Non-alcoholic fatty liver disease (NAFLD) is the accumulation of excessive fat in the liver without any identifiable risk factors, such as alcohol, and is one of the most common causes of chronic liver disease worldwide. NAFLD is the hepatic manifestation of metabolic syndrome. It shares the etiology of systemic inflammation with metabolic syndrome, including obesity, diabetes and dyslipidemia [80]. Studies have shown a direct correlation of NAFLD with various inflammatory biomarkers, such as C-reactive protein [81], interleukins, cytokines and adipokines [82]. Higher levels of Lp-PLA2 were observed in patients with NAFLD compared with healthy individuals, and Lp-PLA2 levels were correlated with the severity of this disease [77]. Metabolic syndrome has been shown to have a poor prognosis in patients with COVID-19. NAFLD is the outcome of visceral adiposity and worsens the metabolic disease. Studies have shown that CVD deaths were the second most common cause of death in patients with NAFLD, and NAFLD/non-alcoholic steatohepatitis (NASH), a state of chronic inflammation due to visceral adiposity, is also a significant risk factor for hospitalization in COVID-19 [83].

## Proposed role of Lp-PLA2 in different pathological stages of COVID-19

### Viral entry & invasion into the lungs

The pathophysiology of COVID-19 starts with SARS-CoV-2 entering the body though the nasal route, which then reaches the alveoli and infects type II alveolar cells. The virus keeps replicating the entire duration of its existence in the body. Affected cells release pro-inflammatory cytokines, and this reaction ultimately affects the immune system [84]. Furthermore, macrophages release interleukins such as IL-1, IL-6 and TNF-α. The release of IL-6 and increased vasodilation facilitate the delivery of more immune cells to the alveolus [85]. Neutrophils release reactive oxygen species and proteinases, leading to increased oxidative stress, which destroys the infected cells and causes tissue injury in the lungs. Therefore, epithelial and endothelial permeability in lungs increases and ultimately a protein-rich fluid accumulates in the alveolus. Finally, the respiratory passage becomes obstructed, which may lead to shortness of breath and the features of pneumonia. The accumulation of fluid in the alveoli and dilution of surfactants lining the alveolus cause alveolar collapse, resulting in hypoxemia and acute respiratory distress syndrome (ARDS). Lp-PLA2 as a good marker for hypoxemic conditions was discussed in the earlier sections; therefore, detecting its levels may be of good prognostic value in COVID-19.

### Cytokine storm

The systemic inflammation and cytokine storm involved in the pathology of COVID-19 can cause septic shock. Therefore, elevated levels of Lp-PLA2 at this point can warn of the imminent risk of cytokine storm and thus the severity of the disease. In COVID-19, knowledge about the etiology and pathophysiology of late complications from acute infection phase is still in its infancy, yet it is known that early and late symptoms are manifestations of a persistent hyper-inflammatory state due to host–viral interactions and inadequate antibody response, which can be better predicted by Lp-PLA2.

### Systemic involvement

Late complications such as endothelitis, myocardial inflammation, ventricular dysfunction, abnormalities in lung function, pulmonary thromboembolism, sleep deregulation and cognitive impairment, though less common, are of poor prognostic value [86]. The derangements in levels of Lp-PLA2 in these conditions were discussed earlier in this article in individual sections. Thus, Lp-PLA2 levels can be used to determine the risk of impending deleterious sequelae among patients with COVID-19. Physical reconditioning at the beginning of the study is also an important predictor of the severity of the disease in infected individuals. Lp-PLA2 has already been recommended for the assessment of risk of certain cardiovascular conditions in otherwise seemingly healthy individuals, which adds to its validity as a biomarker in COVID-19. Hu *et al.* [87] found levels of total cholesterol, HDL-cholesterol and LDL-cholesterol to be significantly decreased in COVID-19 patients. This finding could be explained by lung injury due to reactive oxygen species, which poses a perpetual problem through the formation of cytotoxic and pro-inflammatory byproducts of lipid oxidation. Lungs cover a large surface area, which is directly linked to the external aerobic environment; therefore, any damage to the lungs can result in the generation of oxidative stress events and various lipid modifications. All these events may lead to cancerous cell growth [88]. In SARS-CoV-2-infected patients, increased production of oxidized lipids is observed in injured pneumocytes and macrophages. Thus, oxidized LDL (oxLDL)-trained macrophages interact with a large amount of oxidized lipids in virus-infected areas and utilize oxidized lipids, resulting in short-term lipid depletion. Diabetic patients have been reported to be more prone to experience an inflammatory storm; hence, diabetics are linked to a worse outcome in COVID-19 [89]. Lp-PLA2 levels have also been reported to be increased in admitted stroke patients. The neurobiology of the virus (viral particles get easily detained in amyloid fibrils) leads to neurological impairment in patients with COVID-19, and thus, correlating pathology with increased levels of Lp-PLA2 is quite rational. The association of Lp-PLA2 with NAFLD/NASH has already been reported. Hence, Lp-PLA2 can be used as a reliable biomarker to identify the risk of NAFLD/NASH in COVID-19 patients as well.

Lp-PLA2 has very intricate roles and widespread participation in a range of disorders. Lp-PLA2-targeted therapy in research has long been associated with many controversies. However, at the same time, studies have established Lp-PLA2 as a reliable diagnostic and prognostic marker in vascular inflammation and associated disease complications (Table 6).

**Table 6.** Studies assessing the diagnostic and prognostic roles of Lp-PLA2 in different disease conditions.

Study	System affected	Conclusion	Ref.
Wei *et al.*	Cardio- and cerebrovascular systems	Serum levels of Lp-PLA2 were found to be a predictor of the recurrence of atherosclerosis and ischemic stroke.	[90]
Sertić *et al.*		Lp-PLA2 was postulated as an additional marker in patients with moderate and high risk of cardio- and cerebrovascular events.	[91]
Cucchiara *et al.*		Lp-PLA2 levels were elevated in patients with >50% stenosis. They could give additional predictive information beyond the age, blood pressure, clinical features and duration of transient ischemic attack in clinical risk score.	[92]
Delgado *et al.*		Lp-PLA2 might give noteworthy prognostic data for the early assessment of transient ischemic attack patients.	[93]
Qu *et al.*	Chronic kidney disease	Lp-PLA2 was suggested as a potential prognostic and diagnostic biomarker for chronic kidney disease and carotid atherosclerotic stenosis.	[94]

Furthermore, when COVID-19 patients were treated with Lp-PLA2 inhibitors, analysis revealed the downregulation of glycerol phospholipids and overexpression of lyso phospholipids, as well as free arachidonic acid and oleic acid. The findings ultimately suggested that Lp-PLA2 inhibitors may play a role in COVID-19 [95].

## Conclusion

Although population-based studies have identified various risk factors for a poor prognosis in COVID-19, the clinical course of individual patients infected with the virus is highly variable. However, risk stratification of medical comorbidities and complications as well as the use of surrogate biological markers that predict clinical deterioration would be helpful in timely intervention. Lp-PLA2 has been found to be correlated with most of the known comorbidities and complications associated with the severity of this disease. Hence, well-designed prospective clinical studies may be helpful in establishing the role of Lp-PLA2 as a biomarker in COVID-19 patients.

## Future perspective

COVID-19 has affected millions of people around the world. Regular efforts have been put into different areas of research on the diagnosis, prevention and treatment of the disease. In this literature review, an effort has been made to study a novel biomarker for COVID-19. To our knowledge, a review that emphasizes the use of Lp-PLA2 as a biomarker in the COVID-19 pandemic in risk stratification has not been published to date. Therefore, it is proposed that well-designed prospective clinical studies may be helpful to explore the role of Lp-PLA2 as a biomarker in COVID-19 patients and, once established, will be of great help to humankind.

**Executive summary**

**COVID-19 & expanded research**

- COVID-19 caused by SARS-CoV-2 is affecting both life and the economy all over the world. Mortality due to disease complications makes the picture even more horrifying.
- Various assessment parameters and biomarkers are being tested for the assessment of severity and decisions on the course of treatment. Many population-based studies have identified several risk factors for poor prognosis in COVID-19; however, the clinical course of individual patients infected with the virus is highly variable.
- However, risk stratification of medical comorbidities and complications as well as the use of surrogate biological markers that predict clinical deterioration would be helpful in timely intervention. The identification of new laboratory biomarkers, which will allow classification of the sickness score and help active treatment, is very much needed.

**Lp-PLA2**

- Lp-PLA2 is an enzyme encoded by the *PLA2G7* gene, which circulates in the blood in the form of a complex with low-density lipoprotein and high-density lipoprotein.
- It has been shown to be a good biomarker in patients with various disorders, such as cardiovascular, pulmonary, cerebrovascular and liver disorders and viral infections.

**Lp-PLA2 in COVID-19**

- Lp-PLA2 has been found to be correlated with most known comorbidities and complications associated with the severity of COVID-19.
- Thus, the role of Lp-PLA2 as a biomarker in COVID-19 is worth inspection.
